# Pulmonic Valve Insufficiency After Repair of Tetralogy of Fallot: A Case Report

**DOI:** 10.7759/cureus.107863

**Published:** 2026-04-28

**Authors:** Anthony N Cholagh, Rohan Patel, Kareem Elewa, Yusuf Qadeer, Kellsey Peterson

**Affiliations:** 1 Internal Medicine, Henry Ford Health System, Detroit, USA; 2 Cardiology, Henry Ford Health System, Detroit, USA

**Keywords:** congenital heart disease, pulmonary valve insufficiency, pulmonary valve regurgitation, rv outflow tract, tetralogy of fallot

## Abstract

Repair of Tetralogy of Fallot (TOF) has significantly improved long-term mortality. A consequence of repair is pulmonic valve insufficiency, leading to cardiac remodeling in adulthood, which can often remain asymptomatic for an extended period. This emphasizes the importance of consistent follow-up with an adult congenital heart disease physician to evaluate for these pathophysiologic consequences. We present a case of a young female patient with a history of TOF status post-repair who was lost to follow-up.

## Introduction

Tetralogy of Fallot (TOF) is the most common cyanotic congenital heart disease, and it is characterized by four anatomical defects: (1) ventricular septal defect (VSD), (2) right ventricular (RV) hypertrophy, (3) overriding aorta, and (4) pulmonary stenosis. With advancements in surgical repair of TOF, survival rates have improved, with a survival rate of 97.1% at 10 years and 94.5% at 25 years [1]. Given prolonged survival rates, long-term complications, such as pulmonic valve insufficiency (PVI) and its downstream effects, have become more prevalent. PVI occurs in patients with TOF after surgical repair because the surgeon must disrupt the integrity of the pulmonic valve to relieve the RV outflow obstruction that occurs [2]. Patients with PVI can be asymptomatic for an extended period of time [3]. This ultimately leads to RV volume overload, dilation, and decreased RV function, which can result in heart failure, arrhythmias, and death [4,5].

## Case presentation

A 39-year-old female with a history of TOF, status post-surgical repair at 16 months of age (the type of repair is unclear, as outside hospital records were unavailable), and hypertension presented to one of our satellite hospitals with a chief complaint of bilateral lower extremity edema and dyspnea. The patient reported no cardiology follow-up for over seven years. On admission, she was hypertensive, with a systolic blood pressure exceeding 200 mmHg, requiring initiation of a nitroglycerin drip, which was subsequently weaned off. Auscultation revealed bilateral crackles without an audible murmur. She had 1+ bilateral lower extremity edema. Her B-type natriuretic peptide (BNP) was elevated at 1,230 pg/mL. EKG demonstrated a right bundle branch block. A transthoracic echocardiogram demonstrated an ejection fraction of 58%, a mildly enlarged right ventricle with mild systolic dysfunction, and severe pulmonary regurgitation.

**Figure 1:**
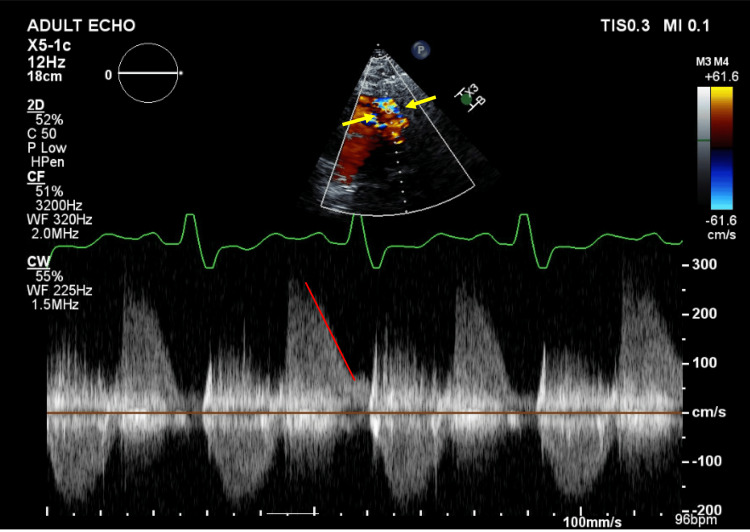
Doppler echocardiogram of the pulmonic valve. The image demonstrates a wide jet width (yellow arrows) and early termination of diastolic regurgitant flow, indicated by a steep slope (red line), indicative of severe pulmonary valve regurgitation. Pressure half-time (PHT) = 111 ms.

**Figure 2:**
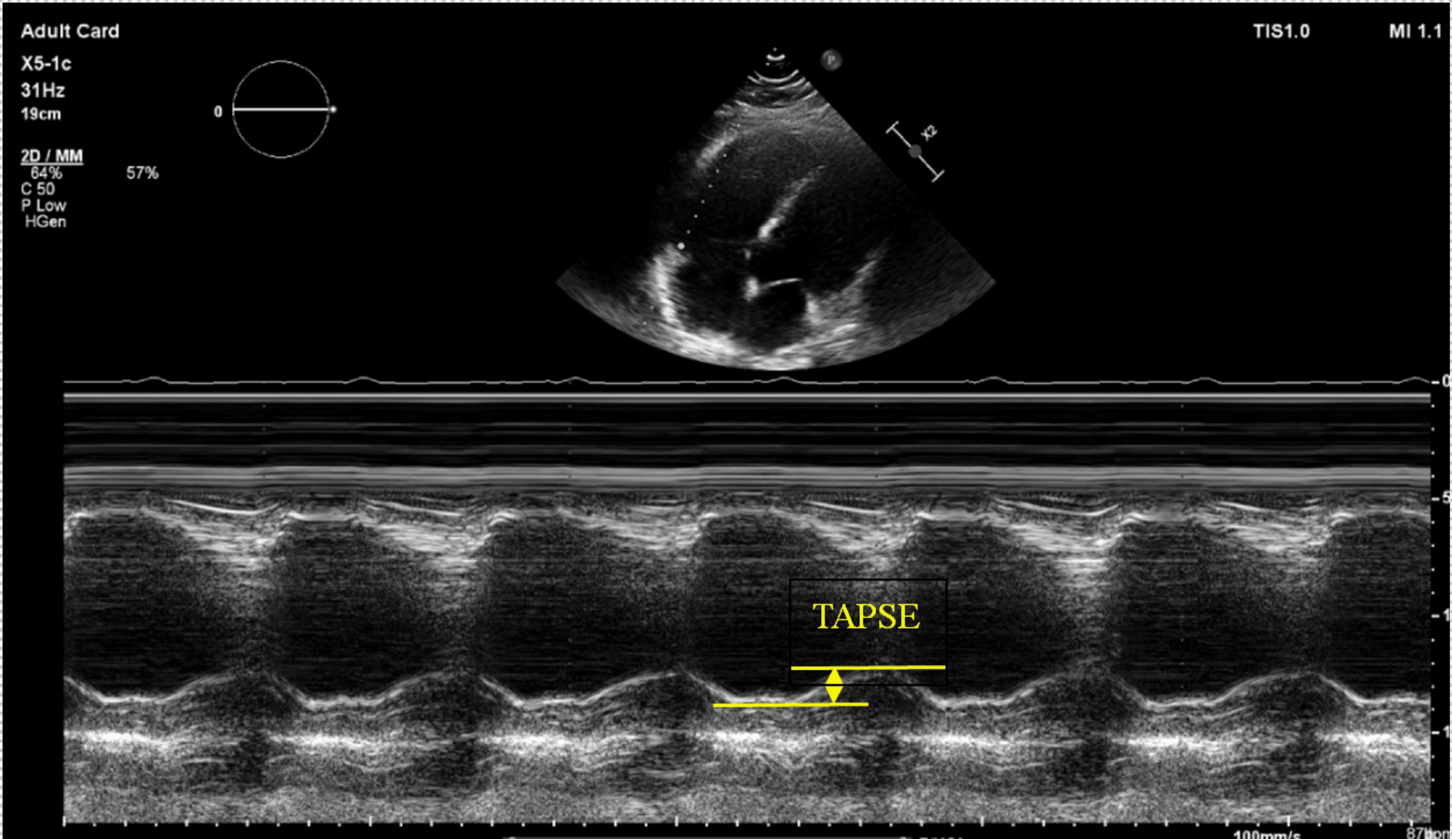
Echocardiogram demonstrating TAPSE. Tricuspid annular plane systolic excursion (TAPSE) is an echocardiographic measure that indicates right ventricular (RV) function. The image above demonstrates a TAPSE of 1.45 cm (normal >1.7 cm), indicating RV dysfunction in this patient.

Given severe pulmonary regurgitation, the patient was transferred to the main hospital for structural heart disease evaluation. A transthoracic echocardiogram with Doppler was obtained, demonstrating a pulmonary regurgitation jet width greater than 70% of the RV outflow tract and a pressure half-time (PHT) of 111 ms, consistent with severe pulmonary regurgitation (Figure 1). She was also noted to have early termination of diastolic regurgitant flow. Tricuspid annular plane systolic excursion (TAPSE) was measured (Figure 2) and was 1.45 cm, indicative of RV systolic dysfunction; values less than 1.7 cm are considered abnormal. Cardiac MRI was obtained to further assess RV function in the setting of pulmonary regurgitation and demonstrated moderate RV dilation with a mildly reduced right ventricular ejection fraction (RV EF) of 36%. RV EF was calculated based on the right ventricular end-diastolic volume (RV EDV) and end-systolic volume (RV ESV), which were 249 mL and 160 mL, respectively. Unfortunately, the MRI was obtained without phase contrast, so a quantitative assessment of the pulmonic valve could not be performed. Given that the patient was clinically stable after treatment of her hypertensive emergency and heart failure exacerbation, the decision was made to arrange outpatient follow-up to evaluate surgical versus transcatheter options for pulmonic valve replacement.

At outpatient follow-up with the structural heart disease team, her symptoms were well controlled with antihypertensive management, so the decision was made to forgo pulmonic valve replacement at this time. She was also evaluated by the adult congenital heart disease team and was advised to undergo a repeat MRI with phase contrast to allow for quantitative assessment of pulmonic valve insufficiency. Once this is completed, a multidisciplinary discussion will be held to determine further management.

## Discussion

Surgical repair of TOF typically involves relieving right ventricular outflow tract obstruction, which often necessitates intervention at the level of the pulmonary valve [6]. This commonly results in chronic pulmonary insufficiency, leading to progressive RV volume overload, RV dilation and dysfunction, and eventual development of tricuspid regurgitation [7,8], as seen in this patient on echocardiogram. Patients develop signs of right-sided heart failure (pedal edema, jugular venous distension) and exercise intolerance [9,10]. This patient presented with signs of RV dysfunction and underwent cardiopulmonary exercise stress testing, which demonstrated exercise intolerance.

Two-dimensional (2D) and Doppler echocardiography are key tools in assessing pulmonary insufficiency. A 2D echocardiogram allows for evaluation of structural indicators of PVI, including right ventricular (RV) and pulmonary artery dilation. Doppler echocardiography allows for assessment of jet width, spectral density of the waveform, and timing of termination of diastolic regurgitant flow. A regurgitant jet width occupying >70% of the RV outflow tract, increased waveform density, and early termination of diastolic regurgitant flow are all consistent with severe pulmonary regurgitation [11].

Treatment of PVI consists of percutaneous versus surgical intervention. Indications for intervention include symptomatic patients with moderate to severe pulmonary regurgitation [11]. A more detailed explanation of indications for pulmonic valve replacement in patients with TOF is shown in Table 1 (adapted from the American Heart Association [12]). Percutaneous pulmonic valve replacement is typically preferred due to similar mortality outcomes compared with surgical intervention and decreased hospital length of stay [13-16]. Surgical valve replacement should be considered in patients whose RVOT anatomy is not suitable for percutaneous intervention or in those with other indications for cardiac surgery [11,14]. Regarding bioprosthetic versus mechanical valves, bioprosthetic valves are preferred, as they avoid the need for anticoagulation and are associated with improved longevity [17].

**Table 1:** Proposed indications for PVR in patients with repaired TOF or similar physiology with moderate or severe PR (regurgitant fraction ≥25%). LV, left ventricular; PR, pulmonary regurgitation; PVR, pulmonary valve replacement; RV, right ventricular; RVOT, right ventricular outflow tract; TOF, Tetralogy of Fallot

Category	Criteria	Details
I. Asymptomatic patients	≥2 of the following criteria:	
	a. RV end-diastolic volume index	>150 mL/m² or *z*-score >4. Alternative: RV/LV end-diastolic volume ratio >2 (when BSA falls outside published normal data)
	b. RV end-systolic volume index	>80 mL/m²
	c. RV ejection fraction	<47%
	d. LV ejection fraction	<55%
	e. Large RVOT aneurysm	Present
	f. QRS duration	>160 ms
	g. Sustained tachyarrhythmia	Related to right-sided heart volume load
	h. Other hemodynamically significant abnormalities:	
		• RVOT obstruction with RV systolic pressure ≥0.7 systemic
		• Severe branch PA stenosis (<30% flow to affected lung) not amenable to transcatheter therapy
		• Greater than or equal to moderate tricuspid regurgitation
		• Left-to-right shunt from residual ASD/VSD with Qp:Qs ≥1.5
		• Severe aortic regurgitation
II. Symptomatic patients	≥1 of the quantitative criteria from Section I, plus:	
	a. Exercise intolerance	Not explained by extracardiac causes. Documentation: ≤70% predicted peak VO₂ for age and sex (not due to chronotropic incompetence)
	b. Heart failure signs/symptoms	Dyspnea with mild effort or at rest, peripheral edema (not explained by extracardiac causes)
	c. Syncope	Attributable to arrhythmia
III. Special considerations		
	a. Late TOF repair	PVR may be considered if ≥1 quantitative criterion from Section I is met. Higher risk in patients repaired at ≥3 years of age
	b. Women of childbearing age	PVR may be considered if ≥1 quantitative criterion from Section I is met. Severe PR with RV dilatation/dysfunction may cause pregnancy complications

## Conclusions

Repair of TOF leads to obligate pulmonic valve insufficiency. The degree of PVI is variable, but if severe, it can lead to downstream consequences of RV volume overload, dilation, arrhythmias, and eventually death. This case highlights the importance of maintaining follow-up in adulthood after repair so that appropriate monitoring for cardiac remodeling and symptoms can be periodically evaluated. In this patient’s instance, she will undergo a repeat Cardiac MRI but will have phased contrast this time to better analyze her degree of pulmonic valve insufficiency.
